# Construction and Analysis of *GmFAD2-1A* and *GmFAD2-2A* Soybean Fatty Acid Desaturase Mutants Based on CRISPR/Cas9 Technology

**DOI:** 10.3390/ijms21031104

**Published:** 2020-02-07

**Authors:** Nan Wu, Qiang Lu, Piwu Wang, Qi Zhang, Jun Zhang, Jing Qu, Nan Wang

**Affiliations:** 1 Jilin Agricultural Science and Technology University, Jilin 132101, China; 2 Jilin Agricultural University, the center of plant biotechnology, Chang Chun 130118, China; zqjlau@163.com (Q.Z.); zhangjunjlnd@163.com (J.Z.); qujing_06@163.com (J.Q.);; 3 Institute of Agricultural College, Jilin Agricultural University, Chang Chun 130118, China; lq329365377@163.com

**Keywords:** soybean oleic acid, FAD2, clustered regularly interspaced short palindromic repeats (CRISPR/Cas9), near-infrared grain analysis

## Abstract

The soybean fatty acid desaturase family is composed of seven genes, but the function of each gene has not been reported. Bioinformatics was used to analyse the structure of genes in this family, as well as the correlation between Δ12-fatty acid desaturase II (FAD2) expression and oleic acid content on different days after flowering of soybean. In the present study, CRISPR/Cas9 technology was used to construct single and double mutant knockout vectors of functional genes in the *FAD2* family. Analysis of the molecular biology and expression patterns of genes in the *FAD2* family, namely, *GmFAD2-1A* (*Glyma.10G278000*) and *GmFAD2-2A* (*Glyma.19G147300*), showed that they had little homology with other soybean *FAD2* genes, and that their function was slightly changed. Sequencing of the target showed that the editing efficiency of the *GmFAD2-1A* and *GmFAD2-2A* genes was 95% and 55.56%, respectively, and that the double mutant editing efficiency was 66.67%. The mutations were divided into two main types, as follows: base deletion and insertion. A near-infrared grain analyser determined the following results: In the T_2_ generation, the oleic acid content increased from 17.10% to 73.50%; the linoleic acid content decreased from 62.91% to 12.23%; the protein content increased from 37.69% to 41.16%; in the T_3_ generation, the oleic acid content increased from 19.15% to 72.02%; the linoleic acid content decreased from 56.58% to 17.27%. In addition, the protein content increased from 37.52% to 40.58% compared to that of the JN38 control variety.

## 1. Introduction

Soybean oil plays an important role in commonly used vegetable oils. According to United States Department of Agriculture (USDA) statistics, the total production of vegetable oil in the world in 2015 amounted to 5.28 million tons, of which 3.2 tons of soybean oil accounted for 55% of the total output share. Soybean oil with high oleic acid content has high antioxidant capacities and stability [1]. Processed soybean oil also has high thermal stability, which can reduce or eliminate chemical hydrogenation and reduce the processing cost [2,3]. Given the continuous growth of the world economy, the consumption demand for soybean oil worldwide will continue to increase. Therefore, the cultivation of soybean varieties with high oleic acid content has become an important goal for soybean breeding.

The most important component of soybean oil is fatty acids, including oleic acid, linoleic acid, linolenic acid, palmitic acid and stearic acid. The synthesis of oleic acid occurs mainly via desaturation by fatty acid desaturase (Δ12-fatty acid desaturase II, FAD2). FAD2 is the key enzyme that determines the composition of linoleic acid, and its activity directly affects the metabolism of oleic acid components and oleic acid in soybean seeds. The accumulation of oleic acid in grain has a direct effect.

The transcription activator-like effector nucleases (TALEN) gene-editing tool has been used for fixed-point editing of the soybean fatty acid dehydrogenase FAD2 family, resulting in increased oleic acid content from 20% to 80% compared to that of wild type [4]. This method has also been utilised to reduce the concentration of linoleic acid, which is harmful to human health, from 50% to 4%, thus improving soybean quality. A combination of mutagenesis and plant tissue culture has been used to cultivate the high-oleic-acid soybean line, USPTO 9198365, which has an oleic acid content as high as 80% [5]. Inhibition of the *FAD2* gene expression in soybean seeds by RNA interference (RNAi) technology increases the oleic acid ratio of soybean oil to more than 85% [6]. Silencing soybean *FAD2* and *FATB* gene expression by RNAi technology greatly increases the oleic acid content in soybeans but decreases the total linoleic acid and linolenic acid content to 3.5% [7]. Many international seed companies, such as Monsanto and DuPont, have also intensified efforts to cultivate new varieties of high-oleic-acid soybeans. The high-oleic-acid Vistiv Gold soybean cultivar produced by Monsanto generates an oleic acid content that is approximately 75% of the total fatty acid content in seed [8]. In addition, DuPont has developed a high-oleic-acid soybean cultivar, named Plenish, with an oleic acid content of 77%, but the linoleic acid and linolenic acid concentrations of this cultivar are only 8% and 3%, respectively [9].

Gene editing can specifically target clustered, regularly interspaced, short palindromic repeats (CRISPRs), and the CRISPR/Cas9 system is an adaptive defence system that exists in bacteria [10,11,12,13]. Using this gene-editing technology, specific target sites are designed, and the *Cas9* protein gene is combined with specific sites to achieve the cleavage and formation of DNA double-strand breaks. The cell itself has repair functions, and the repair mode is divided into nonhomologous end-joining and homologous recombination, which can be used to achieve highly-efficient and directional knockout of a single gene or gene family. This technique has the characteristics of low cost and simple operation [14,15].

Clustered regularly interspaced short palindromic repeats (CRISPR/Cas9) technology has been used to specifically knock out the *CsFAD2* gene in flax, which increases the oleic acid content from 16% to 50% and decreases the linolenic acid and linoleic acid content from 35% to 9% [16]. CRISPR/Cas9 technology has also been used to specifically knock out the *GmWRI1a* gene to study the WRI1 transcription factor, which is involved in fatty acid synthesis and glycolysis pathways, by utilising a near-infrared grain analyser to examine the transgenic strains [17], which determined that the oleic acid content decreases by 11.44% compared to the wild-type material.

## 2. Results

### 2.1. Target Design

Using CRISPR/Cas9 gene-editing technology, the soybean endogenous genes, namely, GmFAD2-1A (g3) and GmFAD2-2A (g6), were knocked out, and the target was designed in the first exon region of the gene using CRISPR-P software as shown in Figure S1.

The sample gRNA target activity was evaluated by comparing it to the two standard gRNA1 (activity 3) and gRNA2 (activity 10), As the estimated activities of the g3 and g6 gRNA were both higher than the standard gRNA2, they were both evaluated as high and suitable for application. According to the instruction of SSA kit, activity3 represents an in vitro digestion activity of 20%, and activity10 represents an in vitro digestion activity of 70%. The results are shown in Figure S2.

Based on greyscale conversion of the enzyme bands by Quantity One software, the calculated gRNA activities are shown in Table S1.

### 2.2. Verification of Expression Vector

The CRISPR/Cas9 knockout vector and universal primers were used for PCR detection (Table S2); the results are shown in Figure S3 (1 and 2 = 350 bp; 3 = 590 bp).

### 2.3. Molecular Detection of Transgenic Offspring

The above knockout vector plasmid was transformed into the JN38 recipient soybean variety, and the T_0_ generation was obtained and analysed by PCR. The following plants were obtained: 20 plants tested positive for pCRISPR/Cas9-g3; 18 plants tested positive for pCRISPR/Cas9-g6; and 42 plants tested positive for pCRISPR/Cas9-g36 (Figure S4).

### 2.4. Sequence Analysis of the Target Mutation of Positive Plants

Specific primers were used to analyse the mutations of two single targets (g3 and g6) and one double target (g36). After PCR amplification, the target fragment was recovered and ligated into the pMD-18T cloning vector. A commercial service performed the sequencing, analysed the editing of the target, integrated the characteristics of the target and compiled the sequencing results (Table 1). The higher the GC content in the target, the harder to be off target [18,19,20,21].

**Figure 1:**
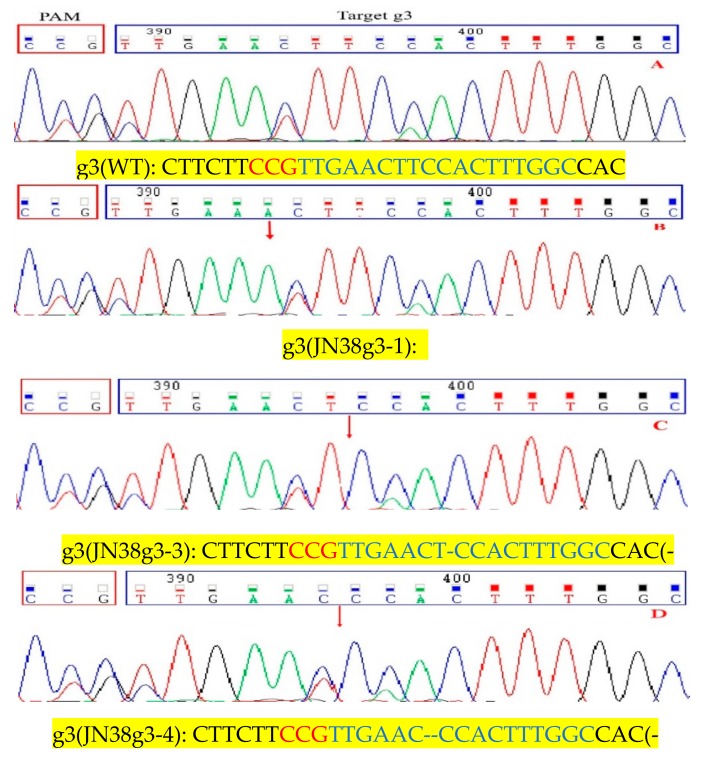
Analyses of sequencing chromatogram data of the target mutant sites of g3. (**A**) Sequences of wild type and representative mutation types induced at target site g3 are presented, respectively. Underline, insertions. Dashes, deletions. (**B**–**D**) are sequence peaks of wild type and representative mutation types at target site g3, respectively. The red arrowheads indicate the location of mutations.

**Figure 2:**
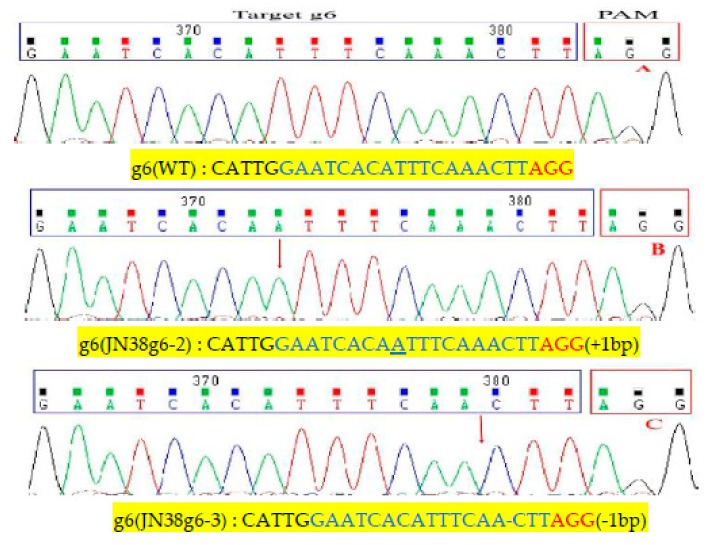
Analyses of sequencing chromatogram data of the target mutant sites of g6. (**A**) Sequences of wild type and representative mutation types induced at target site g6 are presented, respectively. Underline, insertions. Dashes, deletions. (**B**,**C**) are sequence peaks of wild type and representative mutation types at target site g6 respectively. The red arrowheads indicate the location of mutations.

**Figure 3:**
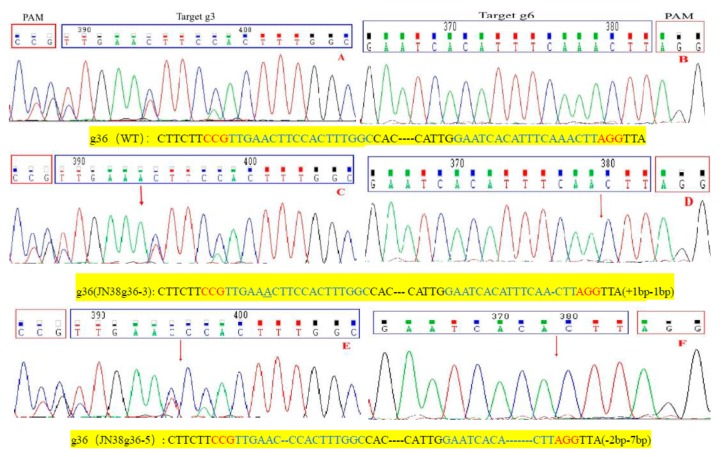
Analyses of sequencing chromatogram data of the target mutant sites of g36. (**A**–**F**) are sequence peaks of wild type and representative mutation types at target site g36 respectively. The red arrowheads indicate the location of mutations.

Further analysis of mutant plants from the T3 generation showed that CRISPR/Cas9 technology produced the following mutation types: homozygous mutations, heterozygous mutations and double allele mutations. In target g3, we selected three homozygous Transfer DNA(T-DNA) free plants (JN38g3-1, JN38g3-3, JN38g3-4), the JN38g3-1 showed common type of homozygous mutation 1 bp insertions, and the proportion of this mutation was approximately 66.7% (Figure 1B). The JN38g3-3 showed common type of mutation was 1 bp deletion, and the proportion of which was approximately 16.6% (Figure 1C). The JN38g3-4 showed proportion of the 2 bp deletion was approximately 16.7% (Figure 1D). For the g6 single target (JN38g6-2, JN38g6-3), the following mutation types were detected: The JN38g6-2 showed common type was 1bp insertion with a proportion was 50% (Figure 2B); and the JN38g6-3 showed type 1bp deletion with a proportion of 50% (Figure 2C). For the g36 double target(JN38g36-3, JN38g36-5), the following mutation types were detected: the JN38g36-3 showed type 1 bp insertion and 1 bp deletion with a proportion of 50% (Figure 3C,D), the JN38g36-5 showed type was 2 bp and 7 bp deletions with a proportion of 50% (Figure 3E,F).

The amino acid sequence of the mutant strains was analysed (Text S2). The results showed that the amino acid sequences of different mutant lines contained deletions or insertions of different lengths, which resulted in frameshifts and advance termination of translation, leading to the gene not being expressed. The above results indicated that the target genes had different degrees of mutation in the transgenic lines. In summary, the homozygous mutants obtained in the present experiment had amino acid frameshift mutations that inactivated the FAD2 soybean fatty acid dehydrogenase family, thereby increasing the oleic acid content of soybean seeds and leading to a new high-oleic-acid soybean germplasm.

### 2.5. Southern Blotting Detection of Positive T3 Plants

**Figure 4:**
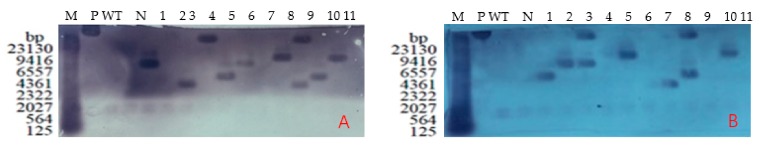
Detection of T_3_ generation positive plants by Southern blotting. (**A**) Target g3 progeny detection; (**B**) Target g6 progeny detection. M: Marker; P: Plasmid; WT: Wild Type; N: Negative signal (trans clean mutant plants).

Genomic DNA was extracted from PCR-positive plants and digested with BamHI and HandIII restriction endonucleases. Bar is suitable as a probe in Southern blotting experiment because it is a marker present in the used construct. As shown in Figure 4, the untransformed plants showed no hybridisation signal, and the transgenic plants with the knockout vectors showed clear hybridisation signals.

### 2.6. Detection of T3 Generation Transgenic Plants by Real-Time PCR

The transgenic plants with SYBR Green I as the dye and positive by Southern blotting were tested by qRT-PCR. As shown in Figure S5, the relative expression levels of *GmFAD2-1A* (g3) and *GmFAD2-2A* (g6) mRNA in mature grains of T_3_ generation transgenic soybeans were significantly lower than those in recipient mature grains. In order to compare WT with mutants, the primer region should not be interested by in/del and mutations. (Error lines are the standard deviation of three replicates).

### 2.7. Determination of Oleic Acid Content in Transgenic T2, T3 Positive Seeds

The oleic acid content of the T_2_ generation transgenic soybean seeds was determined by a near-infrared grain analyser. The oleic acid content in the positive g3 strain increased from 17.10% to 32.11%. In the positive strains with the g6 single target, the oleic acid content increased from 17.10% to 41.35% compared to the content in the Jinong 38 recipient. In the positive g36 double target strain, the oleic acid content increased from 17.10% to 73.50% compared to the content in the Jinong 38 recipient (Table 2).

The oleic acid content of the T_3_ generation transgenic soybean seeds was determined by a near-infrared grain analyser. The oleic acid content in the positive g3 strain increased from 19.15% to 34.47%. In the positive strains with the g6 single target, the oleic acid content increased from 19.15% to 40.45% compared to the content in the Jinong 38 recipient. In the positive g36 double target strain, the oleic acid content increased from 19.15% to 72.02% compared to the content in the Jinong 38 recipient (Table 3).

### 2.8. Significance Analysis of Difference in Oleic Acid Content in Offspring Grains

According to the difference of oleic acid content between different genotypes of T_2_ and T_3_, the results showed that the oleic acid content between the two generations was stable, and the oleic acid content of the offspring of the double target was significantly higher than that of the single target. The acid content indicates that the efficiency of the double target is higher than that of the single target, and the efficiency of the gene *GmFAD2-2A* is higher than that of the gene *GmFAD2-1A* (Table S3).

### 2.9. Analysis of Agronomic Characters of Converted Materials

The transgenic lines and control varieties were investigated under field management (Table 4). Both the JN38 control variety and transgenic lines had white flowers, round leaves and grey hairs. There was no significant difference between the transgenic lines and control varieties. Although the grain size was different, there was a significant positive correlation between 100 grain weight and oleic acid. The average value of the results of three times of 100 grain weight measurement showed that there was no obvious difference in 100 grain weight due to the different density owing to different contents of oleic acid.

### 2.10. Phenotypic Analysis of Genetically Edited Progeny

To analyse the mature phenotype of high oleic acid-positive mutant plants, we selected the CRISPR/Cas9 vector progeny seeds and the receptor JN38 progeny seeds for phenotypic analysis.

**Figure 5:**
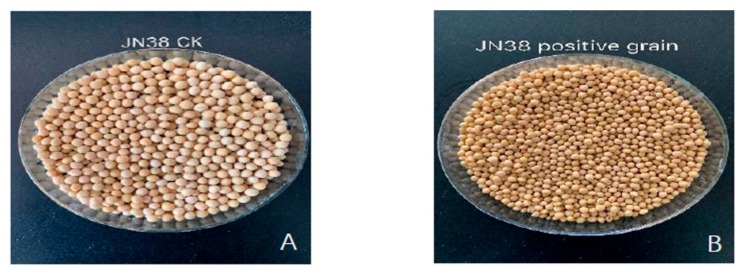
Grain size phenotype analysis. (**A**) JN38 offspring grain phenotype (**B**) Transgenic grain phenotype of CRISPR/Cas9 vector.

The oleic acid content of the progeny of the CRISPR/Cas9 vector was higher, the grain size was significantly smaller, and the colour of the seed coat was deeper, compared to the control variety (Figure 5).

## 3. Discussion

In the process of improving soybean traits, increasing the soybean oleic acid content has always been an important topic of scientific research. The oleic acid content of soybean germplasms in Northeast China is generally between 18.9% and 37.7%, which is considered low [22,23]. Compared to conventional breeding, transgenic breeding can shorten the breeding period, and can be used to directionally cultivate new soybean germplasms. Specific silencing of the *FAD2* soybean fatty acid desaturase gene family by RNAi technology has become increasingly common, and a new high-oleic-acid soybean germplasm has also been obtained [24,25].

The present study used CRISPR/Cas9 technology for the first time to perform fixed-point editing of the *GmFAD2-1A* (g3) soybean fatty acid desaturase gene and its homologous gene, namely, *GmFAD2-2A* (g6), which are regulated by soybean oleic acid synthesis. The vector and bivalent knockout vector showed that higher GC content in the target sequence resulted in greater probability of mutation in plants in the T_0_ generation [26,27]. The editing efficiency of double target is higher than that of single target, because double target is not easy to off-target. Further sequencing analysis showed that a *FAD2* homozygous mutant was obtained in the T_0_ generation. The mutation types were mainly homozygous mutations and heterozygous mutations, and the types of mutations were mainly insertions and deletions of bases, which, in turn, affected the amino acid coding sequences, resulting in protein frameshifts or early termination of expression. By including the T_2_ generation homozygous mutant strain, the *Cas9* gene was detected. Some plants containing no transgenic components were isolated in the T_2_ generation and continued to test positive for the T_2_ homozygous strain. The results showed complete homozygosity, indicating that gene editing was stably inherited by the offspring. The plants carrying the *Cas9* gene in the offspring continued to test positive for the mutation. The mutation type was completely consistent with that of the previous generation, and no new mutation type was produced.

Because soybean is an ancient tetraploid plant, it contains many duplication genes, and the fatty acid desaturase family contains multiple genes, which are related, to jointly regulate the synthesis of oleic acid in soybean. The fatty acid desaturase genes, which are closely related, may be mutated, but these mutants may participate in other metabolic pathways or mobilise the expression of other homologous genes to compensate for the effects of the deletion of these two genes. In addition, determination of the oleic acid content in soybean seeds using a near-infrared grain analyser may result in some errors. However, the oleic acid content was 1% significant different compared to the Jinong 38. The oleic acid content of mature soybean grains in the two generations was slightly different, which may have been due to the influence of environmental and genetic factors during the synthesis of oleic acid in soybean. However, both generations exhibited an upward trend.

There was no significant difference in agronomic traits such as plant height and number of nodes transferred to CRISPR/Cas9 vector-positive plants, but most of the offspring showed variation in grain size. The reason is that the transcriptional regulation of genes is network-like regulation. A gene change may lead to changes in other related metabolic pathways. Although the FAD2 family can regulate soybean oleic acid metabolism, it may also affect the size of soybean grains.

In the present study, CRISPR/Cas9 technology was used for a reverse genetics study of the soybean fatty acid desaturase *GmFAD2* gene, and the results indicated that negative regulation of the *GmFAD2* gene affected the oil content of soybean seeds by modulating the oleic acid content via the two genes. In 2017, Yang et al. Used RNAi technology to silence the *GmFAD2-1B* gene and obtain new soybean germplasm with significantly increased oleic acid content [28]. In 2019, Hou used CRISPR/Cas9 technology to specifically knock out the *GmFAD2-1A* gene and obtain mutant materials whose oleic acid content increased from 20% to 23% [29]. Multiple comparisons showed that the efficiency of the *GmFAD2-2A* gene was higher than that of the *GmFAD2-1A* gene, and high oleic acid mutant with trans clean plants were obtained, which provided a theoretical basis for cultivating high oleic soybeans.

## 4. Materials and Methods

### 4.1. Materials

The Jinong 38 soybean receptor variety, DH5α *Escherichia coli* strain, *Agrobacterium* EHA105 strain and pMD18-T cloning vector were all provided and preserved by the Plant Biotechnology Center of Jilin Agricultural University. The CRISPR/Cas9 Vector Construction Kit was purchased from Hangzhou Baige Biotechnology Co., Ltd (HangZhou, China).

### 4.2. Target Design and Detection of gRNA Target Efficiency by Enzyme Activity in Vitro

Based on the characteristics of the region 18–20 bp upstream of the CRISPR/Cas9 system-specific protospacer adjacent motif, the corresponding targets were designed via the online CRISPR-P tool (v. 2.0) (http://crispr.hzau.edu.cn/CRISPR2/) with the CDS sequences of g3 and g6. Results with high to low scores were obtained, and guide RNA (gRNA) with high fractions and exon regions was selected as the target for subsequent experiments.

The synthetic gRNA-F/gRNA-R primer pair was designed (Table S2), and PCR was performed using the standard gRNA fragment as a template. The PCR system was as follows: 10 ng of gRNA plasmid, 1.5 µL of primer 1, 1.5 µL of primer 2, 25 µL of mix, and diethyl pyrocarbonate (DEPC) H_2_O to a total volume of 50 µL. The PCR procedure was as follows: 3 min at 95 °C, 30 s at 94 °C, 30 s at 58 °C, 30 s at 72 °C, 10 min at 72 °C and 10 min at 16 °C (35 cycles).

Standard gRNA1 primers (g1-FP and gRNA-RP, Table S2) or standard gRNA2 primers (g2-FP and gRNA-RP, Table S2) were used, and PCR was performed using standard gRNA fragments as templates. The PCR system was as described above. The PCR product was approximately 120 bp in length.

The enzyme digestion reaction system was prepared in the order described in Table S4. Each enzyme digestion reaction was performed at the same time as the gRNA digestion reaction for the standard target, and the activity comparison was performed as a positive control (see Section 2.1).

### 4.3. Construction of the CRISPR/Cas9 Expression Vector

Using an in vitro activity detection of >90% of gRNA, online synthesis of the g3UP/g3LOW and g6UP/g6LOW oligo sequences (Table S2) was performed according to the Baige website (www.biogle.cn, February 2017). The synthesised oligo was dissolved in water to 10 µM, and the following reaction system was prepared for oligo dimerisation: 18 µL of annealing buffer, 1 µL of UP oligo and 1 µL of LOW oligo (total volume of 20 µL). After mixing, the reaction was heated at 95 °C for 3 min and then slowly cooled to 20 °C at approximately 0.2 °C/s. The oligo dimer was integrated into a CRISPR/Cas vector using the following reaction system: 6 µL of H_2_O, 2 µL of CRISPR/Cas9 vector, 1 µL of oligo dimer and 1 µL of enzyme mix (total volume of 10 µL). After mixing, the reaction was performed at room temperature (20 °C) for 1 h. Part of the reaction solution (5 µL) was removed and added to *E. coli* competent cells. Construction of the expression vector is shown in Figure S6. The BiogleF/BiogleR vector primer pair (Table S2) was used to verify the sequence of the generated vectors. The plasmids were named pCRISPR/Cas9-g3, pCRISPR/Cas9-g6 and pCRISPR/Cas9-g36 (see Section 2.2).

### 4.4. Detection of Transgenic Plant Progeny

In the present study, the CRISPR/Cas9 plasmid DNA was transferred into the Jinong 38 soybean cultivar by *Agrobacterium*-mediated transformation, and transformed plants were obtained Based on the *Cas9* (662 bp) gene sequence (Text S1), Primer v. 5.0 software was used to design Cas9S/Cas9AS (Table S2). The genomic DNA of T_0_ soybean leaves was extracted using a kit, and the untransformed recipient soybean leaf genome was used as a negative control. The volume of the Cas9 gene PCR system was 25 µL. The following PCR conditions were used: pre-denaturation at 94 °C for 5 min, denaturation at 94 °C for 40 s, renaturation at 60 °C for 40 s and extension at 72 °C for 40 s (30 cycles), followed by a final extension at 72 °C for 8 min. The samples were stored at 4 °C (see Section 2.3).

### 4.5. Editing Efficiency Test

Specific primers, including guide3S/guide3AS and guide6S/guide6AS (Table S2), were used to extract genomic DNA from young leaves of positive plants. After PCR amplification, the product was electrophoresed on a 1% agarose gel to recover the pMD-18T vector, which was transformed into *E. coli* competent cells and sent to Changchun Kumei Biotechnology Co., Ltd. (Chang Chun, China) for sequencing. The mutation type was identified from the sequencing peak map. The sequencing results were analysed by DNAMAN software (v. 6.0) (see Section 2.4.)

### 4.6. Southern Blotting Detection of T_3_ Transgenic Plants

PCR-positive T3 genomic DNA was extracted from the leaves of transgenic plants using the BamHI restriction endonuclease and purified Bar as a probe using the DIG DNA Labeling and Detection Kit I (Roche, Company, Basel, Swiss) according to the manufacturer’s instructions. DNA was used for probe-labelled sample preparation, hybridisation, membrane transfer, membrane cleaning and staining procedures to detect the corresponding Southern blotting hybridization (see Section 2.5).

### 4.7. Quantitative Real-Time PCR detection of T3 Transgenic Plants

RNA from the mature seeds of transgenic plants that tested positive by Southern blotting was extracted using the Total RNA Extraction Kit (Omega Bio-tek, Norcross, GA, USA) and reverse transcribed to obtain cDNA, which was diluted 5-fold. The Qg3/QAg3 and Qg6/QAg6 fluorescent quantitative PCR primers were designed for the target (Table S2). The soybean β-actin gene (GenBank accession number: NM_001252731.2) was selected as the internal reference gene, and the QFACT and QRACT quantitative PCR primers were designed (Table S2). Total RNA from the mature soybean kernels was analysed using a Mx3000P Real-time PCR machine (Jitai Biotech Co., Ltd., Shanghai, China). According to the manufacturer’s instruction for the SYBR Premix Ex TaqTM Kit (Omega Bio-tek, Norcross, GA, USA), the PCR amplification reaction system (25 µL) was set up as follows: 12.5 µL of 2× SYBR Premix Ex Taq polymerase, 1 µL of primer 1, 1 µL of primer 2, 2 µL of template and sterile water for a final volume of 25 µL. The three-step PCR amplification conditions were as follows: pre-denaturation at 95 °C for 3 min, denaturation at 95 °C for 10 s and extension at 60 °C for 35 s (40 cycles (see Section 2.6).

### 4.8. Determination of Oleic Acid Content in T2, T3 Soybean Seeds Using a Near-Infrared Grain Analyser

Near-infrared grain analysis is the most commonly-used spectral analysis technique. In the present experiment, a NIRS DS2500 grain analyser was used to select clean, single, T_2_, T_3_ generation-positive, mature seeds, which were placed in a measuring cup as the seed volumes need to be equal. The bottom of the cup and the edge of the infrared scanning area were placed in the sample tank of the near-infrared grain analyser, and the soybean acid content was determined according to the spectrum acquisition procedure established previously [30,31,32,33,34]. Each sample measurement was repeated three times. The measurement results were collected and analysed by the operation software, and the results were automatically saved to the computer (see Section 2.7).

### 4.9. Significant Analysis of Differences in Oleic Acid Content

We used the SPSS19.0 software (SPSS Inc, Chicago, IL, USA) to analyse the significance of differences in oleic acid content of T2 and T3 soybean seeds by Duncan’s multiple range (see Section 2.8).

### 4.10. Investigation and Phenotypic Analysis of Agronomic Traits in Offspring

Analysis of the agronomic characters of the transformation materials. Under the field management, the transgenic lines and the control varieties were investigated. Compared with the control variety Jinong38, the transgenic lines were all white flowers, round leaves and grey hairs, which had no significant difference with the control varieties (see Section 2.9 and Section 2.10).

## Figures and Tables

**Table 1:** Editing efficiency of each target.

Gene Target	GC%	Edited Efficiency %	Homozygous Mutation Efficiency %
g3	52%	95% (19 plants)	31.58% (6 plants)
g6	33.3%	55.56% (10 plants)	40% (4 plants)
g36	-	66.67% (28 plants)	28.75% (8 plants)

**Table 2:** Changes in fatty acid composition of positive T2 generation strains.

T_2_ Generation Materials	Oleic	Linoleic Acid	Protein	Crude Fat	Oleic Acid Increase Percentage
JN38CK	17.10 ± 0.05	62.91 ± 0.03	37.69 ± 0.05	21.96 ± 0.02	-
g3CRA1	32.11 ± 0.02	48.45 ± 0.05	40.20 ± 0.05	22.66 ± 0.05	87.55
g6CRA1	41.35 ± 0.04	38.81 ± 0.03	40.51 ± 0.03	22.21 ± 0.04	141.5
g36CRA8	73.50 ± 0.02	12.23 ± 0.03	41.16 ± 0.02	20.63 ± 0.03	329.3

**Table 3:** Changes in fatty acid composition of positive T3 generation strains.

T_3_ Generation Materials	Oleic	Linoleic Acid	Protein	Crude Fat	Oleic Acid Increase Percentage
JN38CK	19.15 ± 0.03	56.58 ± 0.02	37.52 ± 0.02	21.02 ± 0.04	-
g3CRA1	34.47 ± 0.02	47.49 ± 0.03	40.58 ± 0.03	22.79 ± 0.03	80.00
g6CRA1	40.45 ± 0.04	41.69 ± 0.04	38.92 ± 0.05	23.92 ± 0.05	111.2
g36CRA8	72.02 ± 0.02	17.27 ± 0.02	39.51 ± 0.03	21.61 ± 0.04	276.1

**Table 4:** Investigation on Agronomic Characters of Transgenic Soybean.

Variety	Plant Height	Section Number	Number of Pods	100-Grain Weight	Yield	Increase Production Ratio
JN38CK	86.6 ± 2.89Aa	15.6 ± 1.08Aa	27.0 ± 3.59Aa	18.32 ± 0.31Aa	0.48 ± 0.04bc	-
g3CRA1	87.2 ± 3.05Aa	16.8 ± 1.75Aa	23.2 ± 4.52Aa	18.85 ± 0.39Aa	0.55 ± 0.02c	14.58
g3CRT5	91.5 ± 3.07Aa	16.5 ± 1.13Aa	22.8 ± 3.81Aa	19.07 ± 0.28Aa	0.47 ± 0.03c	−2.08
g6CRA1	93.4 ± 3.25Aa	14.0 ± 1.19Aa	24.8 ± 4.05Aa	19.33 ± 0.19Aa	0.51 ± 0.07c	6.25
g6CRT5	85.5 ± 2.91Aa	16.3 ± 1.25Aa	25.7 ± 3.69Aa	18.72 ± 0.21Aa	0.58 ± 0.14a	20.8
g36CRA8	92.3 ± 2.65Aa	15.8 ± 1.23Aa	28.2 ± 3.59Aa	19.21 ± 0.12Aa	0.49 ± 0.09c	2.08
g36CRT11	91.8 ± 2.92Aa	16.7 ± 1.05Aa	29.1 ± 3.72Aa	18.85 ± 0.24Aa	0.53 ± 0.05c	10.4

## Supplementary Materials

Supplementary materials can be found at https://www.mdpi.com/1422-0067/21/3/1104/s1. Figure S1—Positions of the genes GmFAD2-1A (A) and GmFAD2-2A (B) and the corresponding targets. Figure S2—(A) From left to right, the lanes are g3, standard gRNA1, standard gRNA2, standard NC, NC, and Marker. (B) From left to right, the lanes are g6, standard gRNA1, standard gRNA2, standard NC, NC, and Marker. Figure S3—CRISPR/Cas9 vector verification. M: DL2000 Marker; 1, 2: single target detection; 3: double target detection. Figure S4—PCR detection of the Cas9 gene (660 bp) in positive plants from the T0 generation. (A) Transfection of the CRISPR/Cas9-g3 vector (partial results). (B) Transfection of the pCRISPR/Cas9-g6 vector (partial results). (C) Transfection of the pCRISPR/Cas9-g36 vector (partial results). Figure S5—Relative gene expression. Figure S6—Structural diagram of the CRISPR/Cas9 expression vector. Table S1—Activity of each gene target. Table S1—primers information. Table S3—Significance Analysis of Oleic Acid Content in Different Generations. Table S4—In vitro digestion system. Text S1—Cas9 DNA sequence. Text S2—Frameshift mutations at two single target sites of GmFAD2-1 and GmFAD2-2b, one double target site generated premature translation termination codons (PTCs).
