# Supplementary material for: Construction and Analysis of GmFAD2-1A and GmFAD2-2A Soybean Fatty Acid Desaturase Mutants Based on CRISPR/Cas9 Technology
Source: Int J Mol Sci. 2020 Feb 7;21(3):1104. doi: 10.3390/ijms21031104 (PMC7037799; doi:10.3390/ijms21031104)
Supplement: Supplementary file 1 [file ijms-21-01104-s001.zip › Supplementary Files/Figure S1.docx]

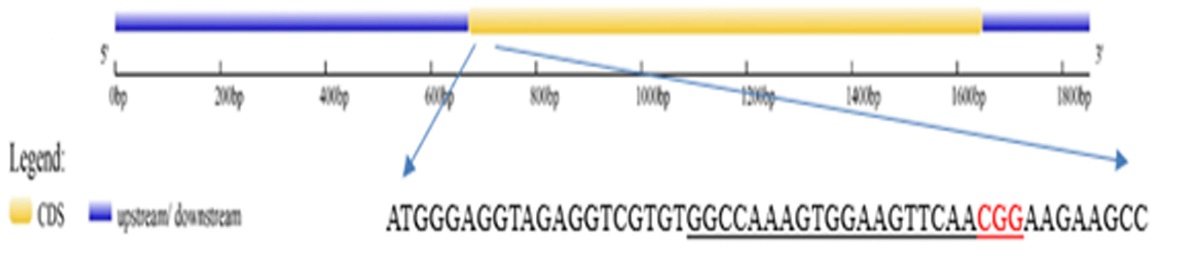


A


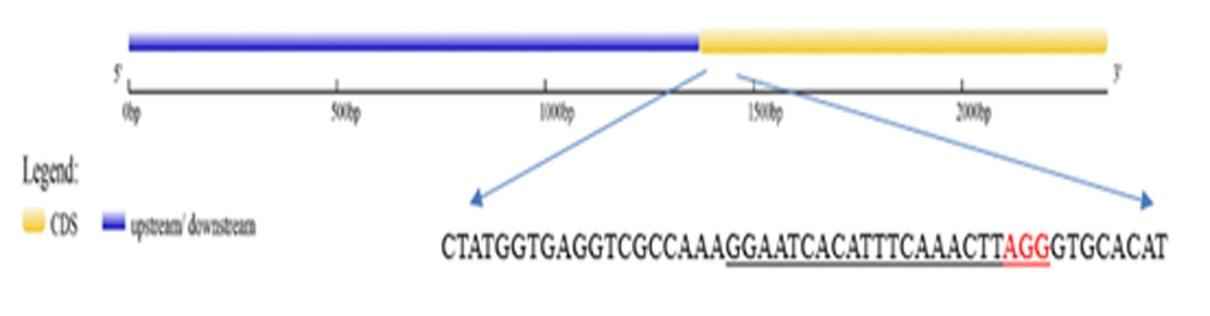


B

**Figure S1.** Positions of the genes *GmFAD2-1A* (A) and *GmFAD2-2A* (B) and the corresponding targets. The upstream and downstream sequences are shown in blue, and the CDS region is shown in yellow.
