# Supplementary material for: Construction and Analysis of GmFAD2-1A and GmFAD2-2A Soybean Fatty Acid Desaturase Mutants Based on CRISPR/Cas9 Technology
Source: Int J Mol Sci. 2020 Feb 7;21(3):1104. doi: 10.3390/ijms21031104 (PMC7037799; doi:10.3390/ijms21031104)
Supplement: Supplementary file 1 [file ijms-21-01104-s001.zip › Supplementary Files/Figure S2.docx]

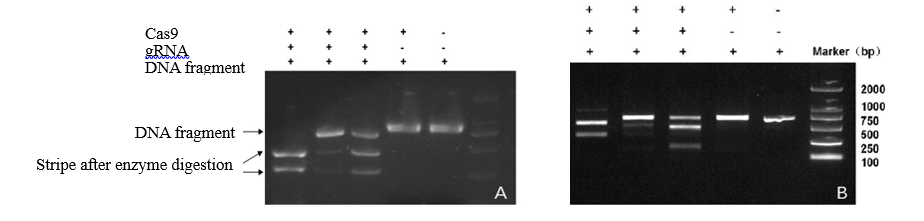
**Figure S2.** (**A**) From left to right, the lanes are g3, standard gRNA1, standard gRNA2, standard NC, NC, and Marker. (**B**) From left to right, the lanes are g6, standard gRNA1, standard gRNA2, standard NC, NC, and Marker.
