# Supplementary material for: Construction and Analysis of GmFAD2-1A and GmFAD2-2A Soybean Fatty Acid Desaturase Mutants Based on CRISPR/Cas9 Technology
Source: Int J Mol Sci. 2020 Feb 7;21(3):1104. doi: 10.3390/ijms21031104 (PMC7037799; doi:10.3390/ijms21031104)
Supplement: Supplementary file 1 [file ijms-21-01104-s001.zip › Supplementary Files/Figure S4.docx]

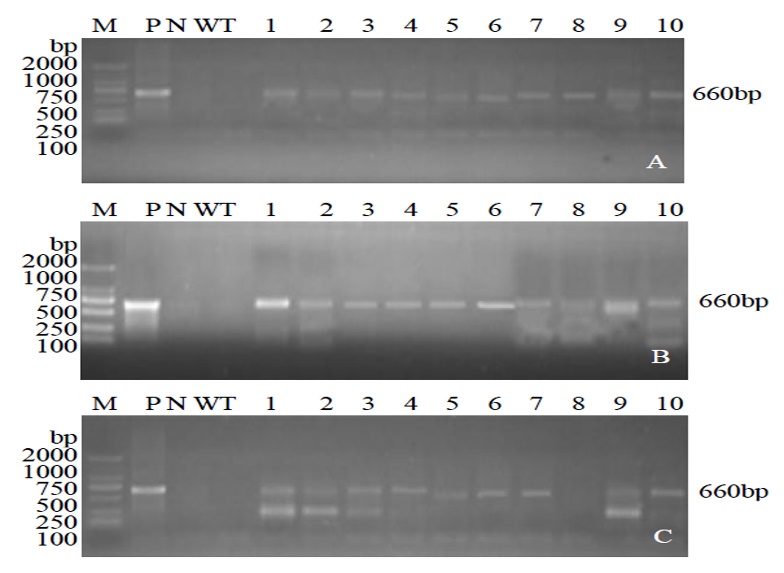


**Figure S4.** PCR detection of the *Cas9* gene (660 bp) in positive plants from the T_0_ generation. (A) Transfection of the CRISPR/Cas9-g3 vector (partial results). (B) Transfection of the pCRISPR/Cas9-g6 vector (partial results). (C) Transfection of the pCRISPR/Cas9-g36 vector (partial results).
