# Supplementary material for: Construction and Analysis of GmFAD2-1A and GmFAD2-2A Soybean Fatty Acid Desaturase Mutants Based on CRISPR/Cas9 Technology
Source: Int J Mol Sci. 2020 Feb 7;21(3):1104. doi: 10.3390/ijms21031104 (PMC7037799; doi:10.3390/ijms21031104)
Supplement: Supplementary file 1 [file ijms-21-01104-s001.zip › Supplementary Files/Table S1.docx]

| **Gene target** | **Target sequence** | **Enzyme activity in vitro** | **Comparison with standard** |
| --- | --- | --- | --- |
| g3 | GGCCAAAGTGGAAGTTCAA**CGG** | 93% | ＞gRNA2 |
| g6 | GGAATCACATTTCAAACTTA**GG** | 95% | ＞gRNA2 |

**Table S1.** Activity of each gene target.
