# Supplementary material for: Construction and Analysis of GmFAD2-1A and GmFAD2-2A Soybean Fatty Acid Desaturase Mutants Based on CRISPR/Cas9 Technology
Source: Int J Mol Sci. 2020 Feb 7;21(3):1104. doi: 10.3390/ijms21031104 (PMC7037799; doi:10.3390/ijms21031104)
Supplement: Supplementary file 1 [file ijms-21-01104-s001.zip › Supplementary Files/Table S2.docx]

| Name | Sequence |
| --- | --- |
| FAD2-2F | CCTGGGTGGTGTGTGTTTAT |
| FAD2-2R | CCCACTCAGAGGAAGTGTAATG |
| FAD2-1bF | CAAAGCCACCCTTCACTCTAA |
| FAD2-1bR | GGTCATAGAGGACGTAGGAGAA |
| FAD2-1F | GGAAACAACAATGGGAGGTAGA |
| FAD2-1R | CAACAGTGAATGGTGGCTTTG |
| FAD2-2aF | CCTGGGTGGTGTGTGTTTAT |
| FAD2-2aR | CCCACTCAGAGGAAGTGTAATG |
| FAD2-2bF | CAGCCAAATCAAGAAGGCTATTC |
| FAD2-2bR | GAGGCAGAAGGCTATGGTAAG |
| FAD2-3F | GTTCTCCGCTCATTCTCCTATG |
| FAD2-3R  FAD2-1BF  FAD2-1BR | GGGAAGGAGGTGGAAGTAATG  CACTCAGAGCTATGGTACTTA  CCACTCAGAGGAAATGGTGAC |
| QFACT | ATCTTGACTGAGCGTGGTTATTCC |
| QRACT | GCTGGTCCTGGCTGTCTCC |
| g3-F | ATGGGAGGTAGAGGTCGTG |
| g3-R | TCAATACTTGTTCCTGTAC |
| g6-F | ATGTGCACCCTAAGTTTGA |
| g6-R | TCACAACTTATTGTTGTAC |
| g3-UP | GGGTTGGGCCAAAGTGGAAGTTCAA |
| g3-LOW | AAACTTGAACTTCCACTTTGGCCCA |
| g6-UP | GGGTTGGGAATCACATTTCAAACTT |
| g6-LOW | AAACAAGTTTGAAATGTGATTCCCA |
| gRNA-F | AGGCACTTACGTCATGGCT |
| gRNA-R | AGCTGCGAGCTAACGTTAA |
| g1-FP | AACTGATGCTTGGCCCAACCGATGCTA |
| g2-FP | AATGCTACGTAGCATTTACGTAACGTTT |
| RNA-RP | AAGTTCCGTACTGGAAATTGCGT |
| BiogleF | CCCAGTCACGACGTTGTAAA |
| BiogleR | CTCGGTGCCACTTTTTCAAG |
| Cas9S | CCCAAGAGGAACAGCGATAAG |
| Cas9AS | GTCGATGGTGGTGTCAAAGT |
| guide3S | ATGGGAGGTAGAGGTCGTGTG |
| guide3AS | GCAACACGGTAGAGAGAGTAAG |
| guide6S | CTGGGAGAGTATTGTACGAAAGTAA |
| guide6AS | CTCTGCCTGGAGGATTGTTAAG |
| Qg3 | CAAAGTGGAAGTTCAACGGAAG |
| QAg3 | GAAAGCAGTGTGGTGGAATTG |
| Qg6 | AGCTCGAGTCGAAGTACTCA |
| QAg6 | AACGGATGAGCCTGATTCTAAC |
| QFACT | ATCTTGACTGAGCGTGGTTATTCC |
| QRACT | GCTGGTCCTGGCTGTCTCC |

**Table S1 primers information**
