# Supplementary material for: Construction and Analysis of GmFAD2-1A and GmFAD2-2A Soybean Fatty Acid Desaturase Mutants Based on CRISPR/Cas9 Technology
Source: Int J Mol Sci. 2020 Feb 7;21(3):1104. doi: 10.3390/ijms21031104 (PMC7037799; doi:10.3390/ijms21031104)
Supplement: Supplementary file 1 [file ijms-21-01104-s001.zip › Supplementary Files/Table S3.docx]

**Table S3. Significance Analysis of Oleic Acid Content in Different Generations.**

| **Variety** | **T_2_** | **Oleic** | **Variety** | **T_3_** | **Oleic** |
| --- | --- | --- | --- | --- | --- |
| JN38 | g3 | 30.50c | JN38 | g3 | 32.18c |
|  | g6 | 40.38b |  | g6 | 39.25b |
|  | g36 | 69.18a |  | g36 | 71.09a |
|  | CK | 17.10d |  | CK | 19.15d |
