# Supplementary material for: Construction and Analysis of GmFAD2-1A and GmFAD2-2A Soybean Fatty Acid Desaturase Mutants Based on CRISPR/Cas9 Technology
Source: Int J Mol Sci. 2020 Feb 7;21(3):1104. doi: 10.3390/ijms21031104 (PMC7037799; doi:10.3390/ijms21031104)
Supplement: Supplementary file 1 [file ijms-21-01104-s001.zip › Supplementary Files/Table S4.docx]

|  | **1** | **2** | **3** |
| --- | --- | --- | --- |
| Cas9 enzyme | 1U | 1U | 1U |
| 10X Cas9 buffer | 2 μl | 2 μl | 2 μl |
| gRNA | 50 ng(sample gRNA) | 50 ng(standard gRNA1(g1)) | 50 ng (standard gRNA2(g2)) |
| ddH_2_O | Xμl | Xμl | Xμl |
| Digested dsDNA | 50 ng | 50 ng( standard DNA) | 50 ng( standard DNA) |
| Total | 20 μl | 20 μl | 20 μl |

**Table S4. In vitro digestion system.**

After thorough mixing, the reaction was carried out at 37 ° C for 0.5 h, and at 65 ° C for 5 min, and the results of the enzyme digestion were analyzed by 1% agarose gel electrophoresis.
