# Supplementary material for: Construction and Analysis of GmFAD2-1A and GmFAD2-2A Soybean Fatty Acid Desaturase Mutants Based on CRISPR/Cas9 Technology
Source: Int J Mol Sci. 2020 Feb 7;21(3):1104. doi: 10.3390/ijms21031104 (PMC7037799; doi:10.3390/ijms21031104)
Supplement: Supplementary file 1 [file ijms-21-01104-s001.zip › Supplementary Files/Text S1.docx]

Text S1.*Cas9* DNA sequence
ATGGCCCCTAAGAAGAAGAGAAAGGTCGGTATTCACGGCGTTCCTGCGGCGATGGACAAGAAGTATAGTATTGGTCTGGACATTGGGACGAATTCCGTTGGCTGGGCCGTGATCACCGATGAGTACAAGGTCCCTTCCAAGAAGTTTAAGGTTCTGGGGAACACCGATCGGCACAGCATCAAGAAGAATCTCATTGGAGCCCTCCTGTTCGACTCAGGCGAGACCGCCGAAGCAACAAGGCTCAAGAGAACCGCAAGGAGACGGTATACAAGAAGGAAGAATAGGATCTGCTACCTGCAGGAGATTTTCAGCAACGAAATGGCGAAGGTGGACGATTCGTTCTTTCATAGATTGGAGGAGAGTTTCCTCGTCGAGGAAGATAAGAAGCACGAGAGGCATCCTATCTTTGGCAACATTGTCGACGAGGTTGCCTATCACGAAAAGTACCCCACAATCTATCATCTGCGGAAGAAGCTTGTGGACTCGACTGATAAGGCGGACCTTAGATTGATCTACCTCGCTCTGGCACACATGATTAAGTTCAGGGGCCATTTTCTGATCGAGGGGGATCTTAACCCGGACAATAGCGATGTGGACAAGTTGTTCATCCAGCTCGTCCAAACCTACAATCAGCTCTTTGAGGAAAACCCAATTAATGCTTCAGGCGTCGACGCCAAGGCGATCCTGTCTGCACGCCTTTCAAAGTCTCGCCGGCTTGAGAACTTGATCGCTCAACTCCCGGGCGAAAAGAAGAACGGCTTGTTCGGGAATCTCATTGCACTTTCGTTGGGGCTCACACCAAACTTCAAGAGTAATTTTGATCTCGCTGAGGACGCAAAGCTGCAGCTTTCCAAGGACACTTATGACGATGACCTGGATAACCTTTTGGCCCAAATCGGCGATCAGTACGCGGACTTGTTCCTCGCCGCGAAGAATTTGTCGGACGCGATCCTCCTGAGTGATATTCTCCGCGTGAACACCGAGATTACAAAGGCCCCGCTCTCGGCGAGTATGATCAAGCGCTATGACGAGCACCATCAGGATCTGACCCTTTTGAAGGCTTTGGTCCGGCAGCAACTCCCAGAGAAGTACAAGGAAATCTTCTTTGATCAATCCAAGAACGGCTACGCTGGTTATATTGACGGCGGGGCATCGCAGGAGGAATTCTACAAGTTTATCAAGCCAATTCTGGAGAAGATGGATGGCACAGAGGAACTCCTGGTGAAGCTCAATAGGGAGGACCTTTTGCGGAAGCAAAGAACTTTCGATAACGGCAGCATCCCTCACCAGATTCATCTCGGGGAGCTGCACGCCATCCTGAGAAGGCAGGAAGACTTCTACCCCTTTCTTAAGGATAACCGGGAGAAGATCGAAAAGATTCTGACGTTCAGAATTCCGTACTATGTCGGACCACTCGCCCGGGGTAATTCCAGATTTGCGTGGATGACCAGAAAGAGCGAGGAAACCATCACACCTTGGAACTTCGAGGAAGTGGTCGATAAGGGCGCTTCCGCACAGAGCTTCATTGAGCGCATGACAAATTTTGACAAGAACCTGCCTAATGAGAAGGTCCTTCCCAAGCATTCCCTCCTGTACGAGTATTTCACTGTTTATAACGAACTCACGAAGGTGAAGTATGTGACCGAGGGAATGCGCAAGCCCGCCTTCCTGAGCGGCGAGCAAAAGAAGGCGATCGTGGACCTTTTGTTTAAGACCAATCGGAAGGTCACAGTTAAGCAGCTCAAGGAGGACTACTTCAAGAAGATTGAATGCTTCGATTCCGTTGAGATCAGCGGCGTGGAAGACAGGTTTAACGCGTCACTGGGGACTTACCACGATCTCCTGAAGATCATTAAGGATAAGGACTTCTTGGACAACGAGGAAAATGAGGATATCCTCGAAGACATTGTCCTGACTCTTACGTTGTTTGAGGATAGGGAAATGATCGAGGAACGCTTGAAGACGTATGCCCATCTCTTCGATGACAAGGTTATGAAGCAGCTCAAGAGAAGAAGATACACCGGATGGGGAAGGCTGTCCCGCAAGCTTATCAATGGCATTAGAGACAAGCAATCAGGGAAGACAATCCTTGACTTTTTGAAGTCTGATGGCTTCGCGAACAGGAATTTTATGCAGCTGATTCACGATGACTCACTTACTTTCAAGGAGGATATCCAGAAGGCTCAAGTGTCGGGACAAGGTGACAGTCTGCACGAGCATATCGCCAACCTTGCGGGATCTCCTGCAATCAAGAAGGGTATTCTGCAGACAGTCAAGGTTGTGGATGAGCTTGTGAAGGTCATGGGACGGCATAAGCCCGAGAACATCGTTATTGAGATGGCCAGAGAAAATCAGACCACACAAAAGGGTCAGAAGAACTCGAGGGAGCGCATGAAGCGCATCGAGGAAGGCATTAAGGAGCTGGGGAGTCAGATCCTTAAGGAGCACCCGGTGGAAAACACGCAGTTGCAAAATGAGAAGCTCTATCTGTACTATCTGCAAAATGGCAGGGATATGTATGTGGACCAGGAGTTGGATATTAACCGCCTCTCGGATTACGACGTCGATCATATCGTTCCTCAGTCCTTCCTTAAGGATGACAGCATTGACAATAAGGTTCTCACCAGGTCCGACAAGAACCGCGGGAAGTCCGATAATGTGCCCAGCGAGGAAGTCGTTAAGAAGATGAAGAACTACTGGAGGCAACTTTTGAATGCCAAGTTGATCACACAGAGGAAGTTTGATAACCTCACTAAGGCCGAGCGCGGAGGTCTCAGCGAACTGGACAAGGCGGGCTTCATTAAGCGGCAACTGGTTGAGACTAGACAGATCACGAAGCACGTGGCGCAGATTCTCGATTCACGCATGAACACGAAGTACGATGAGAATGACAAGCTGATCCGGGAAGTGAAGGTCATCACCTTGAAGTCAAAGCTCGTTTCTGACTTCAGGAAGGATTTCCAATTTTATAAGGTGCGCGAGATCAACAATTATCACCATGCTCATGACGCATACCTCAACGCTGTGGTCGGAACAGCATTGATTAAGAAGTACCCGAAGCTCGAGTCCGAATTCGTGTACGGTGACTATAAGGTTTACGATGTGCGCAAGATGATCGCCAAGTCAGAGCAGGAAATTGGCAAGGCCACTGCGAAGTATTTCTTTTACTCTAACATTATGAATTTCTTTAAGACTGAGATCACGCTGGCTAATGGCGAAATCCGGAAGAGACCACTTATTGAGACCAACGGCGAGACAGGGGAAATCGTGTGGGACAAGGGGAGGGATTTCGCCACAGTCCGCAAGGTTCTCTCTATGCCTCAAGTGAATATTGTCAAGAAGACTGAAGTCCAGACGGGCGGGTTCTCAAAGGAATCTATTCTGCCCAAGCGGAACTCGGATAAGCTTATCGCCAGAAAGAAGGACTGGGACCCGAAGAAGTATGGAGGTTTCGACTCACCAACGGTGGCTTACTCTGTCCTGGTTGTGGCAAAGGTGGAGAAGGGAAAGTCAAAGAAGCTCAAGTCTGTCAAGGAGCTCCTGGGTATCACCATTATGGAGAGGTCCAGCTTCGAAAAGAATCCGATCGATTTTCTCGAGGCGAAGGGATATAAGGAAGTGAAGAAGGACCTGATCATTAAGCTTCCAAAGTACAGTCTTTTCGAGTTGGAAAACGGCAGGAAGCGCATGTTGGCTTCCGCAGGAGAGCTCCAGAAGGGTAACGAGCTTGCTTTGCCGTCCAAGTATGTGAACTTCCTCTATCTGGCATCCCACTACGAGAAGCTCAAGGGCAGCCCAGAGGATAACGAACAGAAGCAACTGTTTGTGGAGCAACACAAGCATTATCTTGACGAGATCATTGAACAGATTTCGGAGTTCAGTAAGCGCGTCATCCTCGCCGACGCGAATTTGGATAAGGTTCTCTCAGCCTACAACAAGCACCGGGACAAGCCTATCAGAGAGCAGGCGGAAAATATCATTCATCTCTTCACCCTGACAAACCTTGGGGCTCCCGCTGCATTCAAGTATTTTGACACTACGATTGATCGGAAGAGATACACTTCTACGAAGGAGGTGCTGGATGCAACCCTTATCCACCAATCGATTACTGGCCTCTACGAGACGCGGATCGACTTGAGTCAGCTCGGGGGGGATAAGAGACCAGCGGCAACCAAGAAGGCAGGACAAGCGAAGAAGAAGAAGTAG
