# Supplementary material for: Construction and Analysis of GmFAD2-1A and GmFAD2-2A Soybean Fatty Acid Desaturase Mutants Based on CRISPR/Cas9 Technology
Source: Int J Mol Sci. 2020 Feb 7;21(3):1104. doi: 10.3390/ijms21031104 (PMC7037799; doi:10.3390/ijms21031104)
Supplement: Supplementary file 1 [file ijms-21-01104-s001.zip › Supplementary Files/Text S2.docx]

**Text S2**：Frameshift mutations at two single target sites of *GmFAD2-1* and *GmFAD2-2b*, one double target site generated premature translation termination codons (PTCs)

CDS of *GmFAD2-1*（g3）wild type:

ATGGGAAAGAACAATAGTATTTCTTATATAGGCCCATTTATGTTGCAAACAATCTTCAAAAGTCCCACATCGCTTATATACGAAAACGAAGCTGAGTTTATATACAGCTACAGTCGAAGTAGTGAGTGGCCAAAGTGGAAGTTCAACGGAAGAAGCTACAAATATCAAGTTAGAATATGGCTAGTCCGTTATCAACTTCAAAAAGTGGCACCGAGTCGGTGCTTTTTTTACTATTTTTGA

Protein sequence of *GmFAD2-1*（g3）wild type:

**Met** G K N N S I S Y I G P F **Met** L Q T I F K S P T S L I Y E N E A E F I Y S Y S R S S E W P K W K F N G R S Y K Y Q V T I W L V R Y Q L Q K V A P S R C F F Y Y F **Stop**

g3(1 bp insertion) CDS

ATGGGAAAGAACAATAGTATTTCTTATATAGGCCCATTTATGTTGCAAACAATCTTCAAAAGTCCCACATCGCTTATATACGAAAACGAAGCTGAGTTTATATACAGCTACAGTCGAAGTAGTGAGTGGCCAAAGTGGAAGTTTCAACGGAAGAAGCTACAAATATCAAGTTAGAATATGGCTAGTCCGTTATCAACTTCAAAAAGTGGCACCGAGTCGGTGCTTTTTTTACTATTTTTGA

Protein sequence

**Met** G K N N S I S Y I G P F **Met** L Q T I F K S P T S L I Y E N E A E F I Y S Y S R S S E W P K W K F Q R K K L Q I S S **Stop**

g3(1 bp deletion) CDS

ATGGGAAAGAACAATAGTATTTCTTATATAGGCCCATTTATGTTGCAAACAATCTTCAAAAGTCCCACATCGCTTATATACGAAAACGAAGCTGAGTTTATATACAGCTACAGTCGAAGTAGTGAGTGGCCAAAGTGG-AGTTCAACGGAAGAAGCTACAAATATCAAGTTAGAATATGGCTAGTCCGTTATCAACTTCAAAAAGTGGCACCGAGTCGGTGCTTTTTTTACTATTTTTGA

Protein sequence

**Met** G K N N S I S Y I G P F **Met** L Q T I F K S P T S L I Y E N E A E F I Y S Y S R S S E W P K W S S T E E A T N I K L E Y G **Stop**

g3(2 bp deletion) CDS

ATGGGAAAGAACAATAGTATTTCTTATATAGGCCCATTTATGTTGCAAACAATCTTCAAAAGTCCCACATCGCTTATATACGAAAACGAAGCTGAGTTTATATACAGCTACAGTCGAAGTAGTGAGTGGCCAAAGTGG--GTTCAACGGAAGAAGCTACAAATATCAAGTTAGAATATGGCTAGTCCGTTATCAACTTCAAAAAGTGGCACCGAGTCGGTGCTTTTTTTACTATTTTTGA

Protein sequence

**Met** G K N N S I S Y I G P F **Met** L Q T I F K S P T S L I Y E N E A E F I Y S Y S R S S E W P K W V Q R K K L Q I S S **Stop**

CDS of *Gm FAD2-2b*（g6）wild type:

ATGGGTGGCAACATAATAGAGGTAGAAGGCTATGGTGAGGTCGCCAAAGGAATCACATTTCAAACTTCGGGTGCACATTTAACATTCCAAACCAATTCAAGACAAATAATATCATTAGCAATTTATAACTGTTGGGGAAAGACGAAACATTGTGCAAACAATTCCAAAAAAAAAATAAAAATGGAATGCTACAAAAAAAATATTACTAATAAATTGTTTAATTGA

Protein sequence:

**Met** G G N I I E V E G Y G E V A K G I T F Q T S G A H L T F Q T N S R Q I I S L A I Y N C W G K T K H C A N N S K K K I K **Met** E C Y K K N I T N K L F N **Stop**

g6：(1bp insertion) CDS

ATGGGTGGCAACATAATAGAGGTAGAAGGCTATGGTGAGGTCGCCAAAGGAATCACAATTTCAAACTTCGGGTGCACATTTAACATTCCAAACCAATTCAAGACAAATAATATCATTAGCAATTTATAACTGTTGGGGAAAGACGAAACATTGTGCAAACAATTCCAAAAAAAAAATAAAAATGGAATGCTACAAAAAAAATATTACTAATAAATTGTTTAATTGA

Protein sequence:

**Met** G G N I I E V E G Y G E V A K G I T I S N F G C T F N I P N Q F K T N N I I S N L **Stop**

g6:(1bp deletion) CDS

ATGGGTGGCAACATAATAGAGGTAGAAGGCTATGGTGAGGTCGCCAAAGGAATCACATTTCAA-CTTCGGGTGCACATTTAACATTCCAAACCAATTCAAGACAAATAATATCATTAGCAATTTATAACTGTTGGGGAAAGACGAAACATTGTGCAAACAATTCCAAAAAAAAAATAAAAATGGAATGCTACAAAAAAAATATTACTAATAAATTGTTTAATTGA

Protein sequence:

**Met** G G N I I E V E G Y G E V A K G I T F Q L R V H I **Stop**

g36（1bp insertion＋1bp deletion）：

ATGGGAAAGAACAATAGTATTTCTTATATAGGCCCATTTATGTTGCAAACAATCTTCAAAAGTCCCACATCGCTTATATACGAAAACGAAGCTGAGTTTATATACAGCTACAGTCGAAGTAGTGAGTGGCCAAAGTGGAAGTTTCAACGGAAGAAGCTACAAATATCAAGTTAGAATATGGCTAGTCCGTTATCAACTTCAAAAAGTGGCACCGAGTCGGTGCTTTTTTTACTATTTTTGA--------------------

ATGGGTGGCAACATAATAGAGGTAGAAGGCTATGGTGAGGTCGCCAAAGGAATCACATTTCAA-CTTCGGGTGCACATTTAACATTCCAAACCAATTCAAGACAAATAATATCATTAGCAATTTATAACTGTTGGGGAAAGACGAAACATTGTGCAAACAATTCCAAAAAAAAAATAAAAATGGAATGCTACAAAAAAAATATTACTAATAAATTGTTTAATTGA

Protein sequence：

**Met** G K N N S I S Y I G P F **Met** L Q T I F K S P T S L I Y E N E A E F I Y S Y S R S S E W P K W K F Q R K K L Q I S S **Stop** ------------------

**Met** G G N I I E V E G Y G E V A K G I T F Q L R V H I **Stop**

g36（2bp deletion＋7bp deletion）：

ATGGGAAAGAACAATAGTATTTCTTATATAGGCCCATTTATGTTGCAAACAATCTTCAAAAGTCCCACATCGCTTATATACGAAAACGAAGCTGAGTTTATATACAGCTACAGTCGAAGTAGTGAGTGGCCAAAGTGG—GTTCAACGGAAGAAGCTACAAATATCAAGTTAGAATATGGCTAGTCCGTTATCAACTTCAAAAAGTGGCACCGAGTCGGTGCTTTTTTTACTATTTTTGA---------------------

ATGGGTGGCAACATAATAGAGGTAGAAGGCTATGGTGAGGTCGCCAAAGGAATCACA-------CTTCGGGTGCACATTTAACATTCCAAACCAATTCAAGACAAATAATATCATTAGCAATTTATAACTGTTGGGGAAAGACGAAACATTGTGCAAACAATTCCAAAAAAAAAATAAAAATGGAATGCTACAAAAAAAATATTACTAATAAATTGTTTAATTGA

Protein sequence

**Met** G K N N S I S Y I G P F **Met** L Q T I F K S P T S L I Y E N E A E F I Y S Y S R S S E W P K W V Q R K K L Q I S S **Stop---------------------**

**Met** G G N I I E V E G Y G E V A K G I T L R V H I **Stop**
